# Graphene oxide electrodeposited electrode enhances start-up and selective enrichment of exoelectrogens in bioelectrochemical systems

**DOI:** 10.1038/s41598-017-14200-7

**Published:** 2017-10-23

**Authors:** R. M. Alonso, M. I. San-Martín, A. Sotres, A. Escapa

**Affiliations:** 1 Chemical and Environmental Bioprocess Engineering Group, Natural Resources Institute (IRENA), Universidad de León, Avda. de Portugal 41, Leon, 24009 Spain; 2 Department of Electrical Engineering and Automatic Systems, Universidad de León, Campus de Vegazana s/n, 24071 León, Spain

## Abstract

This study seeks to assess the impact that the anodic electrodeposition of graphene oxide (GO) has on the start-up process and on the development of microbial communities on the anode of BESs. The GO electrodeposited electrodes were characterised in abiotic conditions to verify the extent of the modification and were then transferred to a bioelectrochemical reactor. Results showed that the modified electrode allowed for a reduced start-up time compared to the control electrode. After three months, high throughput sequencing was performed, revealing that electrochemically reduced graphene oxide acts as a selective agent toward exoelectrogenic bacteria as *Geobacter*. Overall, this study shows that GO modified electrodes enhance biofilm build up in BES.

## Introduction

**Figure 1:**
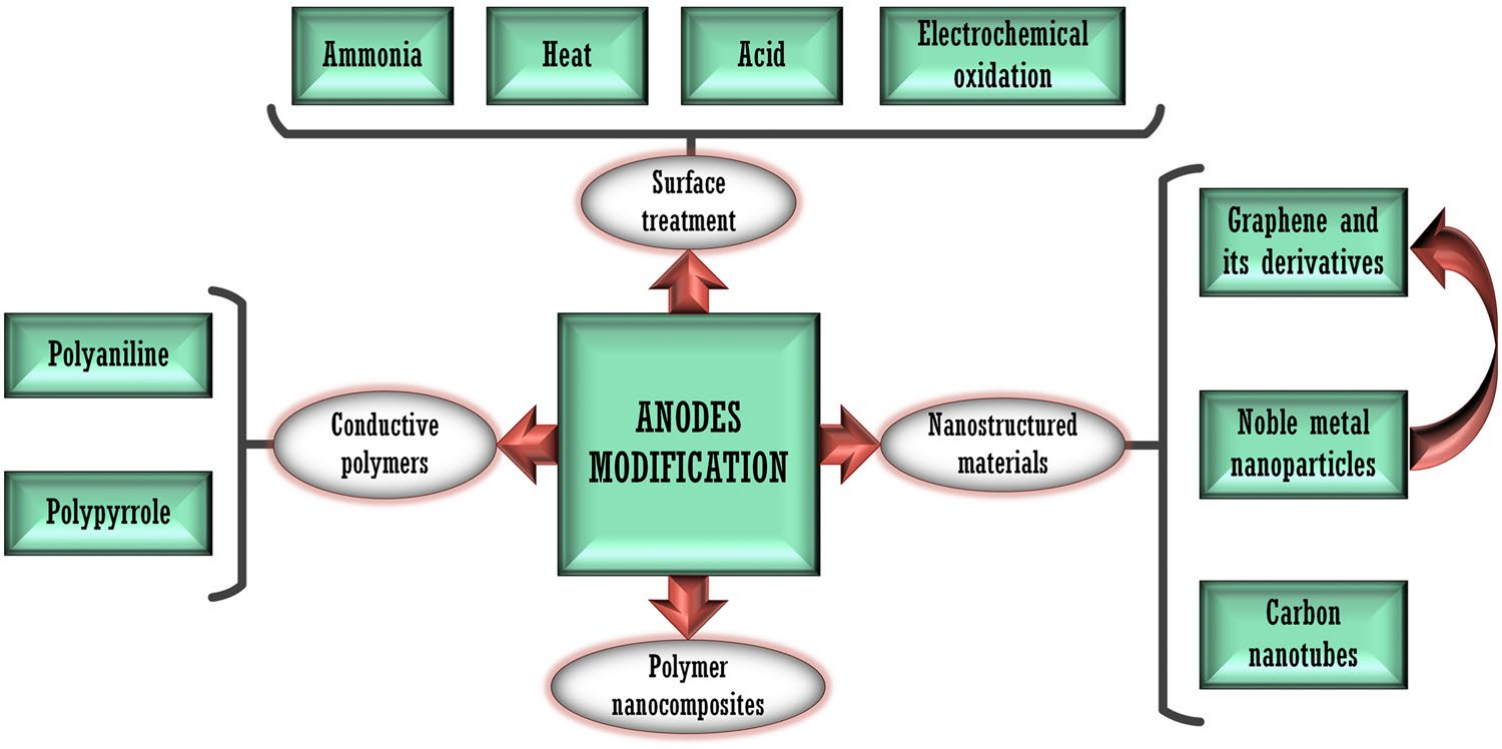
Schematic representation of different strategies used for the modification of BES anodes.

Practical implementation of BES technologies still demands a considerable effort to make this technology economically and technically competitive. Process efficiencies, and particularly current densities usually found at laboratory and pilot scale, are not suitable for an industrial application. This has led to important efforts in new materials development, searching a compromise between high efficiency and low cost features. Nonetheless, engineering, electrochemical and biological factors as well as process control strategies still have much to contribute to make these technologies advance. As proof of this is the increasing momentum that the modification of biofilm–electrode interfaces (usually with small biocompatible molecules and nouvelle materials^1,2^) has gained in recent years, allowing for improving key performance parameters in bioelectrochemical systems (BES), such us biofilm development time, current density or mass transfer between the bulk solution and the exoelectrogenic biofilm. Figure 1 summarises some of the modification methods already tested in BES anodes (for more detailed information, we refer the reader to the excellent reviews of Kumar *et al*.^3^ and Liu *et al*.^4^). The present study can be considered part of the ‘nanostructured materials’ branch, in which the electrode is modified with complex carbon molecules that possess metallic or semiconductor properties, which can participate in charge transfer processes^5^.

Graphene derivatives represent an interesting group of suitable nanostructured materials for electrode modification due to their unique characteristics, such as large specific surface area, high electrical conductivity, chemical stability and electro-catalytic activity, for a variety of redox reactions. These aspects have led to an intensive application of graphene modified materials in the electrochemistry field, especially in sensing and energy storage, and generation areas^6^.

Several studies have been published on the application of graphene in BES, especially in microbial fuel cells. Apart from the advantages previously discussed, these studies have also reported the positive effect of graphene derivatives over the biofilm build-up and the interactions between the material and the microorganisms^7,8^. However, the antibacterial effect of graphene-based materials is still a controversial issue. Some studies point out the lack of antibacterial effects of the GO^9^, while on the contrary, other studies affirm that the GO materials destroy cellular integrity^10^. Furthermore, to date, most of the studies have been mostly restricted to assess the interactions of pure strains with graphene^9,11,12^, while there is still a lack of knowledge about the effect of these graphene-based materials on the overall microbial community of a mixed culture.

To our knowledge, the present study provides, for the first time, an effective method for GO electrodeposition on a carbon brush electrode (anode) via an electro-reductive process based on a conventional potentiostatic technique. This represents a fast and effective method that avoids the use of contaminant reagents, and presents a low sensitivity to temperature and reduced stoichiometry uncertainty^13^, thus providing a sustainable and technically sound alternative to methods like thermal, chemical, photothermal and laser-induced reduction methods. The GO electrodeposited electrodes were characterised in abiotic and biotic conditions, and bacterial communities on the anode biofilm were analysed by means of high throughput sequencing at the end of the experiment. Our experiments were performed using an environmental inoculum in order to gain insights about how a real and high diversity inoculum responds to a graphene modified electrode. Above all, we aim at assessing both the effect of electrochemically reduced GO (erGO) on the start-up process of a bio-anode, and how the erGO impacts on the development of the microbial communities in the anodic biofilm after a long-term operation of the BES.

## Materials and Methods

### Preparation of graphene brush modified electrode

Graphene oxide (GO) was electrodeposited on a carbon brush electrode under deoxygenated conditions (guaranteed via nitrogen bubbling) through a series of 16 cyclic voltammetries (CV) between −1.5 and 0.8 V vs. Ag/AgCl (3 M) and at a scan rate of 20 mVs^−1^. The electrodes consisted of carbon fibers (PANEX 35 50 K, Zoltek) wound into two twisted titanium wires (length = 95 mm, diameter = 0.8 mm, gauge 20), provided by Mill-Rose (US). The brush length was 2 cm and the diameter, 1 cm. The minimum potential selected for carrying out the simultaneous reduction-electrodeposition process (−1.5 V) was chosen to maximise the reduction of oxygen-based groups, avoiding the damage of deposited graphene film caused by hydrogen evolution^13^. The brush was connected as a working electrode (WE) and a platinum wire mesh (2 cm × 2 cm, Goodfellow, UK) served as counter electrode (CE). The electrolyte consisted of an aqueous solution containing 150 mM NaCl and 0.5 mgmL^−1^ of GO (TheGrapheneBox, Spain). The solution was previously neutralised with 0.5 M KOH and then sonicated for 15 minutes. The GO dispersion used contained 4 mg·mL^−1^ of GO in nanosheet form and was prepared according to modified Hummers method^14^ (further information about this reagent can be obtained from www.thegraphenebox.com). The graphene modified brush will be referred to as electrochemically reduced GO electrode (erGO electrode) throughout the manuscript.

### Electrodes characterisation

The microstructures of erGO and unmodified electrodes was examined by the JEOL JSM-6480LV scanning electron microscope (SEM). The fibres of the electrodes were cautiously cut up and rinsed in deionized water and dried at ambient temperature. Prior to observation of the microstructure by SEM, the fibres were sprayed with a thin layer of gold with a Leica EM ACE600 equipment. Fourier-transform infrared spectroscopy (FTIR) was performed in order to search for changes in the electrodes chemical properties. FTIR spectra of the samples were recorded by a Thermo IS5 Nicolet spectrophotometer and acquired from 400 to 4000 cm^−1^ at room temperature (16 scans and spectral resolution of 4 cm^−1^). The samples for FTIR analysis were prepared by cutting and subsequently fine grinding in a mixer ball mill (MM200, Retsh, Germany) the fibres of the electrodes. The peak positions were determined using Origin 2015 software.

### Electrochemical analysis

The abiotic characterisation of the electrodes was performed using a BioLogic VSP potentiostat. The data plot and analysis were carried out using the software associated with the equipment (EC-Lab® V10.40). The ohmic drop is compensated by an built-in method in the EC-Lab® software, based on resistance determination through Electrochemical Impedance Spectroscopy (EIS). A 100 mL conical cell (Metrohm 6.1415.210), using a three-electrode configuration with an Ag/AgCl reference electrode (RE) (Bioblock Scientific) and a platinum mesh (Goodfellow) were used as CE. The reaction medium was previously sparged for 20 minutes with pure nitrogen to remove dissolved oxygen that interferes in the cyclic voltammetry.

### MECs set-up and operation

The biotic characterisation of the electrodes was performed on single chamber MEC reactors (50 mL volume), and were constructed according to Call and Logan^15^. The single chamber design was chosen due to its simplicity and advantageous reproducibility^16^. The anodes of the (MECs) were inoculated by mixing river mud obtained from a local river with growth medium in a 1:5 volume ratio. The growth medium composition per litre was 0.87 g of K_2_HPO_4_, 0.68 g of KH_2_PO_4_, 0.25 g of NH_4_CL, 0.453 g of MgCl2·6H2O, 0.1 of KCl, 0.04 of CaCl2·2H2O, 10 mL of mineral solution and 500 mgL^−1^ of sodium acetate were added to the mixture as carbon source. The mineral solution composition is detailed in Marshall *et al*.^17^. The cathodes were made with stainless steel mesh (AISI304) and their area was approximately 5 cm^2^.

MECs were operated in fed-batch mode at 30 °C under controlled temperature conditions. A fresh medium solution with acetate was refilled when the current dropped below 100 μA. The cell potential was poised at 1 V between anode and cathode.

### Microbial community analysis

In order to study the microbial community attached to the electrodes, two different samples from each graphite brush were cut at different points. Genomic DNA from these electrodes were extracted with the PowerSoil® DNA Isolation Kit (MoBio Laboratories Inc., Carlsbad, CA, USA), following the manufacturer’s instructions. The entire DNA extract was used for the pyrosequencing of the 16S-rRNA gene-based massive library targeting the eubacterial region V1-V3 16S-rRNA and performed at MR DNA (www.mrdnalab.com, Shallowater, TX, USA) utilising MiSeq equipment (Illumina, San Diego, CT, USA). The primer set used was 27Fmod (5′-AGRGTTTGATCMTGGCTCAG-3′) /519 R modBio (5′-GTNTTACNGCGGCKGCTG-3′). Diluted DNA extracts were used as a template for PCR reactions. The obtained DNA reads were compiled in FASTq files for further bioinformatics processing. Trimming of the 16S-rRNA bar-coded sequences into libraries was carried out using QIIME software version 1.8.0^18^. Quality filtering of the reads was performed at Q25 quality prior to grouping into Operational Taxonomic Units (OTUs) at a 97% sequence homology cut-off. The following steps were performed using QIIME: a denoising procedure by using a denoiser algorithm^19^. Final OTUs were taxonomically classified using BLASTn against a database derived from the Ribosomal Database Project II (RDPII, http://rdp.cme.msu.edu) and the National Centre for Biotechnology Information (NCBI, www.ncbi.nlm.nih.gov). Raw pyrosequencing data obtained from this analysis were deposited in the Sequence Read Archive (SRA) of the NCBI under nucleotide sequence accession numbers SRP117204.

To evaluate the diversity of the samples, microbial richness estimators (observed OTUs and Chao1) and diversity indices estimators (Shannon (H’) and 1/Simpson) were calculated with the defined OTUs table (shared.file) using MOTHUR software, version 1.35.1 (http://www.mothur.org), for each sample^20^, and normalising the number of reads of all samples with those of the sample with the lowest number of reads. The genus chord diagram was made using the statistical software R, version 3.3.2 (http://www.R-project.org)^21^.

## Results and Discussion

### Electrochemical behaviour of the erGO electrode

**Figure 2:**
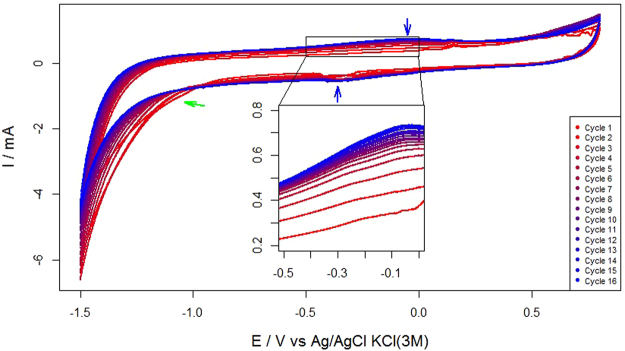
Cyclic voltammetry curves obtained during 16 cycles of simultaneous reduction/electrodeposition electrode modification procedure. The blue arrows point to the reduction/oxidation peaks attributed to oxygen-based functionalities. The green arrow shows the beginning of the hydrogen evolution reaction.

The original brush electrode was modified by a series of CV following the method described above (section: Preparation of graphene brush modified electrode). The CV profiles (Fig. 2) tended to move upwards in the positive direction of the Y-axis (see the inset in Fig. 2) as the number of cycles increased, which indicates that the capacitance of the electrode increased with every new cycle, thus confirming that a conductive film had been electrodeposited over the working electrode. The relevance of the electrode capacitance in BES performance has been reported in several studies^22,23^.

All CVs clearly exhibited a cathodic wave starting at −1.0 V (green arrow), which is generally attributed to the irreversible electrochemical reduction of GO^24^ following the reaction Eq. (1):

1 $$GO+x{H}^{+}+y{e}^{-}\to erGO+z{H}_{2}O$$

This process is responsible for transforming nonconductive GO into electrochemically-reduced GO (erGO), which is a good electron conductor^25^. Other less apparent reduction and oxidation peaks (approximately placed at −0.4 and −0.1 V, blue arrows) could arise due to the activity of oxygen-based functional surface groups^26^. Interestingly, the presence of such remaining oxygen-based functionalities seems to be related with surface electrode properties like hydrophilicity, which may influence biofilm formation and development^27^.

**Figure 3:**
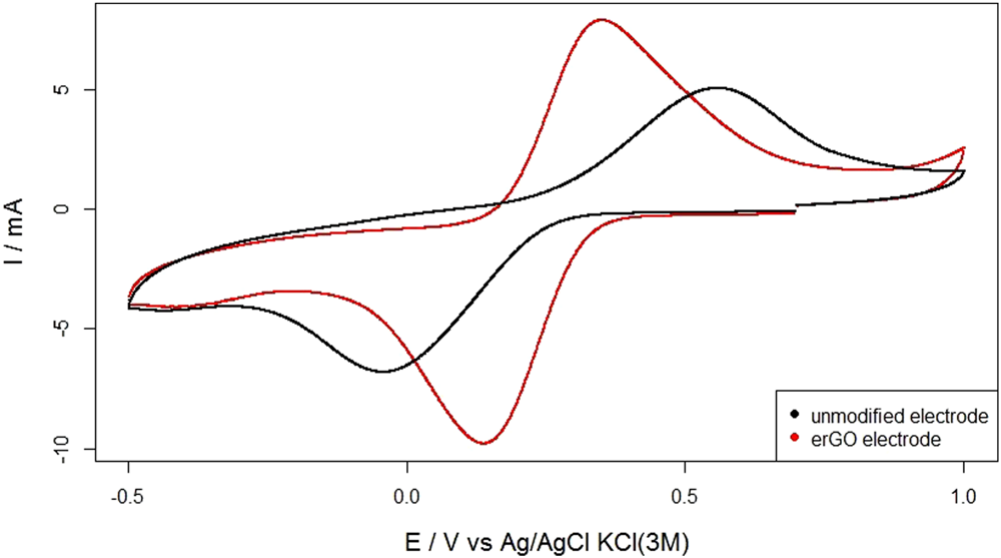
Cyclic voltammetry performed at a scan rate of 20 mV·s^−1^ in 3.4 mM K3Fe(CN)6 and 0.1 M KCl as supporting electrolyte for the unmodified and erGO electrodes.

To evaluate the impact of the GO electrodeposition on the electrochemical properties of the electrode, several CVs were performed in a solution containing 3.4 mM K_3_Fe(CN)_6_ and 0.1 M KCl as supporting electrolyte (for both the unmodified or control and erGO electrodes). The voltammograms (Fig. 3) were recorded at 20 mVs^−1^ scan rate, revealing two clear-cut redox waves corresponding to the K_3_Fe(CN)_6_/K_4_Fe(CN)_6_ pair. The higher peak corresponded to the erGO modified electrodes (7.6 against 4.2 mA referring to the correspondent baselines^28^), which points to enhanced electrocatalytic activity (attributable to a larger electroactive surface area), thus confirming the results outlined in Fig. 2. Moreover, the peak-to-peak separation was much smaller in the erGO electrode (599 versus 210 mV), revealing an electrochemical behaviour much closer to reversibility (at least for the K_3_Fe(CN)_6_/K_4_Fe(CN)_6_ pair). All these observations indicate that the deposited graphene enhanced the heterogeneous electron transfer between the solution and the solid electrode surface.

### Assessing the microstructure and chemical composition of the erGo electrode

**Figure 4:**
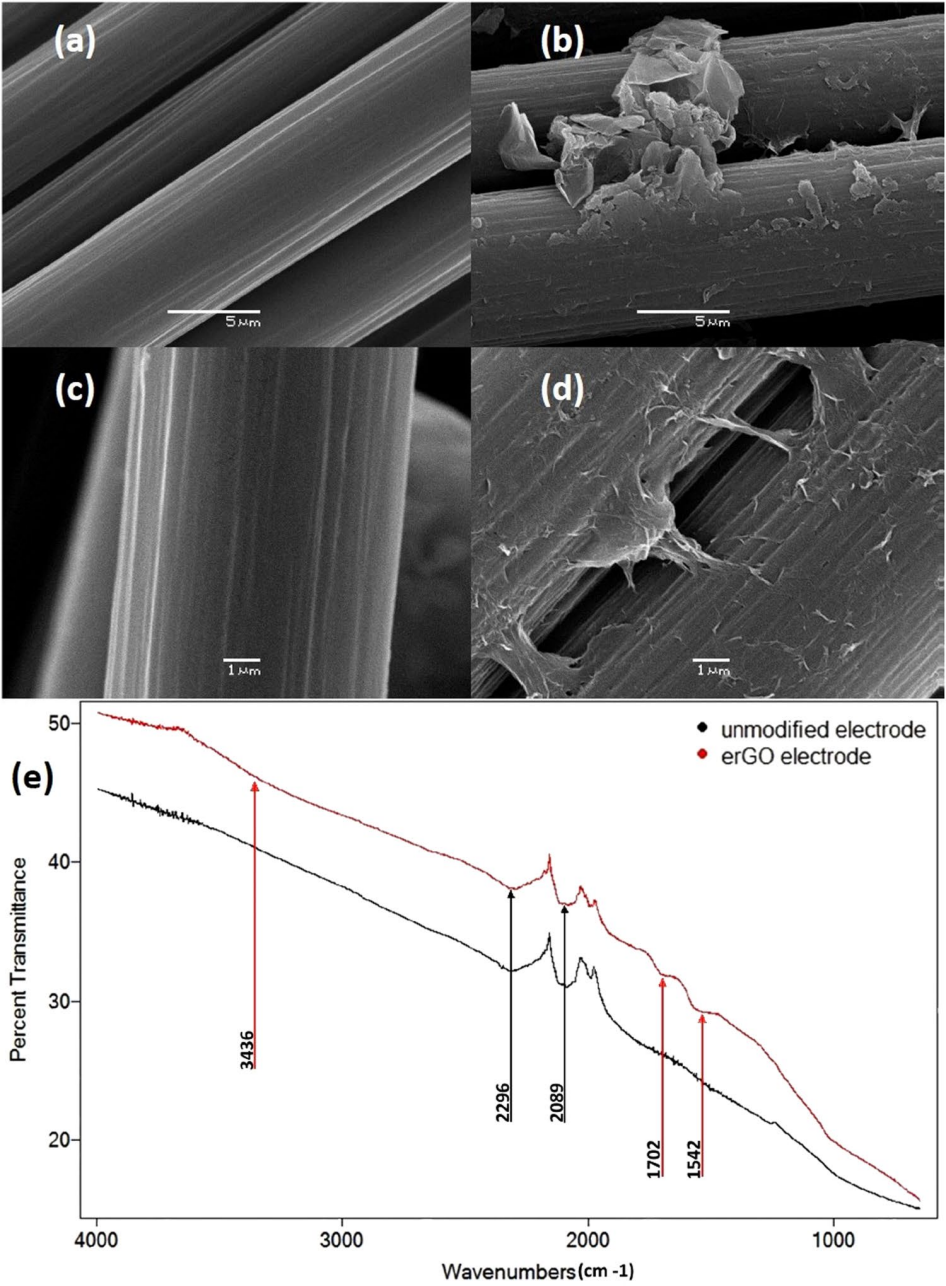
SEM images of electrode fibers. (**a**) unmodified X5000 magnification. (**b**) erGO X5000 magnification. (**c**) unmodified X10000 magnification. (**d**) erGO X10000 magnification. (**e**) FTIR spectra of unmodified (black) and erGO (red) electrode fibers.

To confirm that the electrochemical behavior of the erGO electrode can be confidently attributed to the presence of electro-reduced graphene, SEM and FTIR spectroscopy analyses were performed. Figure 2a–c shows SEM images of the modified and unmodified electrodes fibers. While the surface morphology of the unmodified fibers exhibits a characteristic striated pattern, the modified fibers appeared coated with a layered film that can be attributed to the electro reduced graphene. Moreover, the film often stretches between adjacent fibers (Fig. 4d), a fact that could contribute to enhance the conductivity of the overall electrode.

Figure 4e represents the FTIR spectra for both, the unmodified and erGO electrodes. Black arrows mark the peaks attributable to the support material (the graphite fibers) while the red arrows mark the peaks related to the erGO modification. Maxima near 2296 cm^−1^ and 2089 cm^−1^ correspond to the presence of unsaturated nitrile groups, and were present in both samples. The peaks at 3436 cm^−1^ and 1702cm^−1^ are characteristic of the epoxy and carbonyl groups in the erGO electrode arrangement, that were referred as oxygen-based functionalities in the electrochemical characterization of the erGO electrode. The 1542 cm^−1^ peak in erGO electrode sample could be attributed to the skeletal vibration of non-oxidized graphite, although this last point is unclear.

### Assessing the bioelectrochemical behaviour of the erGO electrode

**Figure 5:**
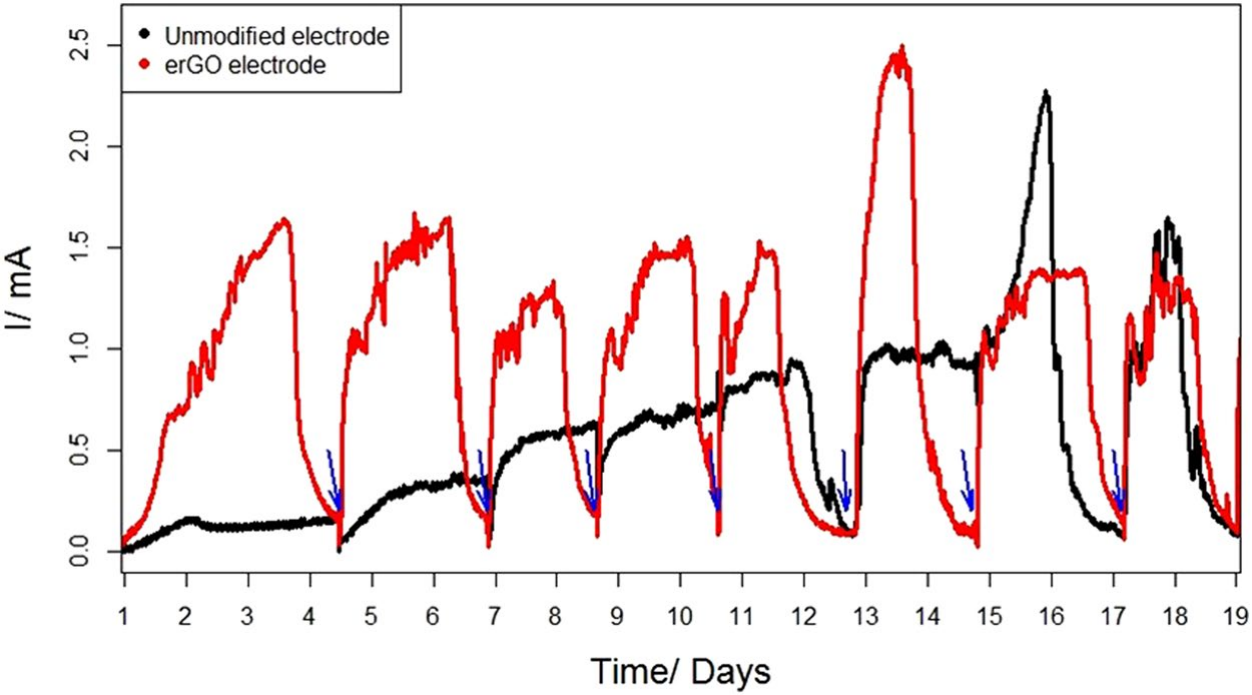
Chronoamperometries obtained for the erGO and unmodified electrodes after inoculation. The lag phase for the unmodified electrodes (10 days) is not included. Blue arrows represent the point where growth medium was replaced.

Following its abiotic characterisation, the erGO electrode was transferred to an MEC and was operated as the anode to assess its behaviour in a bioelectrochemical environment. A similar MEC was used as a control (with an unmodified anode) and both the erGO and the unmodified electrode MECs were inoculated using the same inoculum and following the same procedure. Significant differences in the performance of the erGO-MEC and control were observed from the moment of inoculation. One of the most striking contrasts is related to the start-up period; while the erGO electrode MEC needed about 30 hours (Fig. 5), the control MEC (unmodified electrode) required approximately ten days (the 10-day lag phase for the control MEC is not represented in Fig. 5), which could be indicative of favourable conditions for bacterial adhesion on the erGO electrode^7^. Moreover, the exponential current increase typically found in BES during the start-up presented a much steeper slope for the erGO electrode, and the erGO MEC achieved a steady current in a matter of five days, while the MEC with the unmodified electrode needed about 14 days (Fig. 5). These facts can be related to a biofilm enhancer role of graphene-based materials, as already observed by other authors^8,29^.

Despite the differences concerning the start-up, the electrical currents produced by the erGo and the MEC with the unmodified electrode tended to converge over time. The average current over a period of six months of operation was 0.94 mA for the unmodified electrode MEC and slightly higher (1.12 mA) for the erGO. Moreover, acetate was totally consumed in both reactors, as it was confirmed from organic and inorganic carbon analysis at the end of every cycle feed. This convergence in the reactors performance contrasts with the considerable differences observed between the communities attached to the electrodes (see the microbial communities analysis section), which suggests the presence of a limiting factor for current production other than the microbial environment (e.g. biofilm maximum density or a limiting diffusion of protons out of the biofilm).

### Bacterial communities structure

This section tries to clarify to what extent the GO electrodeposition affected to the microbial diversity and the biofilm development in our set-up. Diversity indices and rarefaction analyses were performed for both anodic biofilms (erGO and unmodified electrodes). As can be seen in Table 1, diversity indices (Shannon (H’) and 1/Simpson index) were only slightly higher in the erGO electrode (H’ = 2.5, 1/Simpson = 3.9) compared to unmodified electrode (H’ = 1.8, 1/Simpson = 3.1). However, species richness indicators (observed OTUs and Chao1 estimator) differed significantly between unmodified and erGO electrode, with the latter scoring the highest indications.

**Table 1:** Number of sequences and operational taxonomic units (OTUs), estimated richness (observed OTUS and Chao1), and diversity indices (Shannon (H’) and 1/Simpson) for eubacterial OTUs.

Sample	No. seqs.	Observed OTUs	Chao1		Shannon (H’)		1/Simpson	
			mean	(c.i.)*	mean	(c.i.)*	mean	(c.i.)*
**Unmodified electrode**	86468	111	166.7	136.8–231.1	1.8	1.82–1.84	3.1	3.06–3.13
**erGO electrode**	435633	659	664.2	659.9–686.6	2.5	2.48–2.50	3.9	3.87–3.01

^*^c.i. 95% confidence intervals.

**Figure 6:**
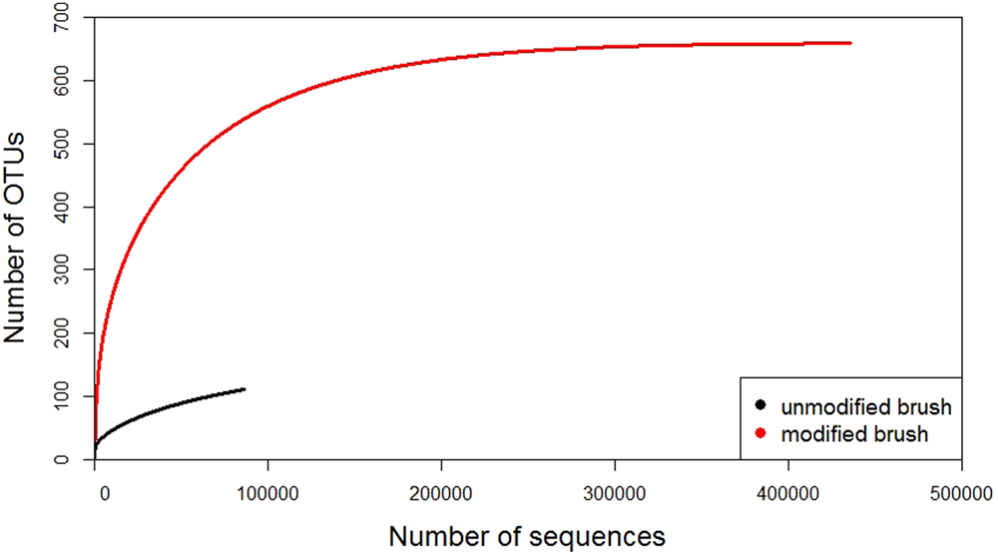
Rarefaction curves of eubacterial number of operational taxonomic units (OTUs) obtained from high throughput sequencing results, for the unmodified and erGO electrodes.

Furthermore, the number of quality reads in the erGO electrodes (435,633) is five times higher than that of the unmodified electrode (86,468) as observed from Table 1 and rarefaction curves (Fig. 6), which implies a greater biomass proliferation in the erGO electrode. These results suggest that the modification in the erGO electrode led to an enhanced bacterial attachment, thus accelerating the biofilm formation^30^ and speeding-up the start-up process – all of which could help to explain the differences observed in Fig. 5. An increase in the hydrophilicity of the erGO electrode (derived from the GO electrodeposition) might be the cause of these differences^26,31^.

**Figure 7:**
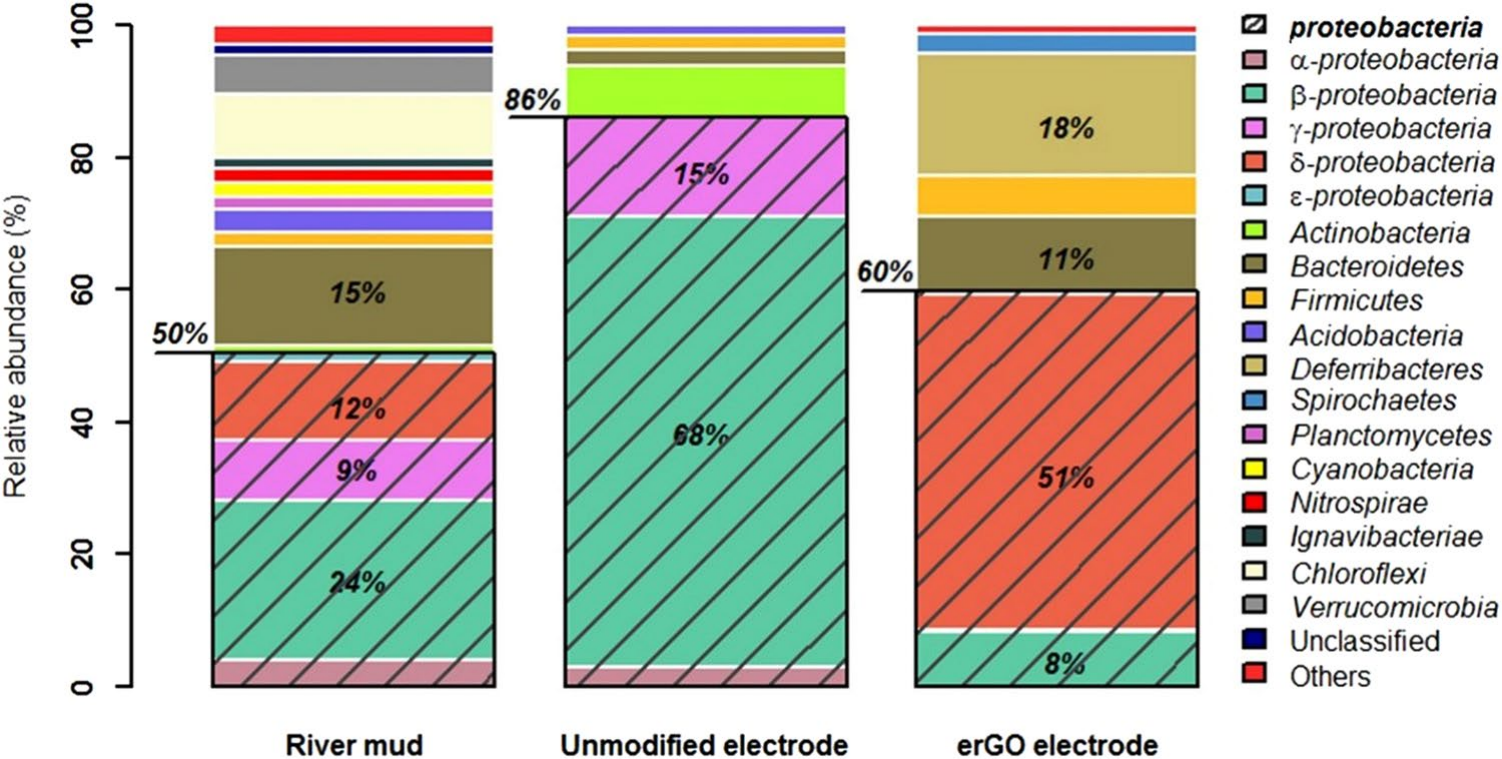
Taxonomic assignment of high throughput sequencing data from eubacterial communities of the initial inoculum (river mud) and of both electrodes (unmodified and erGO electrodes) at a phylum level. Groups accounting for less than 1% of the total number of sequences per sample were classified as ‘others’.

To better understand the effect of the GO electrodeposition over the structure of the microbial communities initially present in the inoculum, a taxonomy assignment was performed. The river mud sample, used as inoculum for both reactors, showed a very high diversity represented by 12 different phyla (Fig. 7). The microbial population was primarily composed of bacteria from the phylum *Proteobacteria* (50%), members of phylum *Bacteroidetes* (15%), and followed by many phyla represented in lower relative abundances as *Chloroflexi* (10%), *Verrucomicrobia* (6%), *Acidobacteria* (4%), and *Firmicutes* (2%) among others. After an acclimatisation period (4 months) in both MEC reactors, two aspects deserve consideration regarding the anodic microbial communities: (i) the diversity decreases drastically in the anodic biofilms, and (ii) a different selective enrichment is observed in the unmodified electrode compared to the erGO electrode. In both electrodes, the eubacterial community was also primarily composed of microorganisms from phylum *Proteobacteria*, 86% and 60% in unmodified and erGO electrode, respectively. However, in the unmodified electrode, *β-proteobacteria* is the predominant class of *Proteobacteria* (68%), mainly represented by the *Burkholderiaceae* family. However, the best part of the *Proteobacteria* phylum in the erGO electrode belongs to the class *δ-proteobacteria* (51%), where the *Geobacteraceae* family represents 50% of the sequences of this class. Members of phylum *Bacteroidetes* are also found in both electrodes – 2% and 11% in unmodified and erGO electrode, respectively. Contrary to members of the phylum *Proteobacteria*, which have been described as playing an important role for the extracellular electron transfer (EET) in the biofilms^32^, *Bacteroidetes* do not have a clear function in electricity generation. However, this phylum is often found abundant in the biofilm consortium, suggesting, therefore, that they could also be important for efficient biofilm function^32^. *Deferribacteres* only found in erGO electrode (19%) is a phylum of anaerobic iron-reducing bacteria and phylogenetically close to the *Geobacter* cluster; hence, they could have a relevant role in MEC performance. Interestingly, this phylum has also been identified in other reduced graphene oxide electrodes^33^.

**Figure 8:**
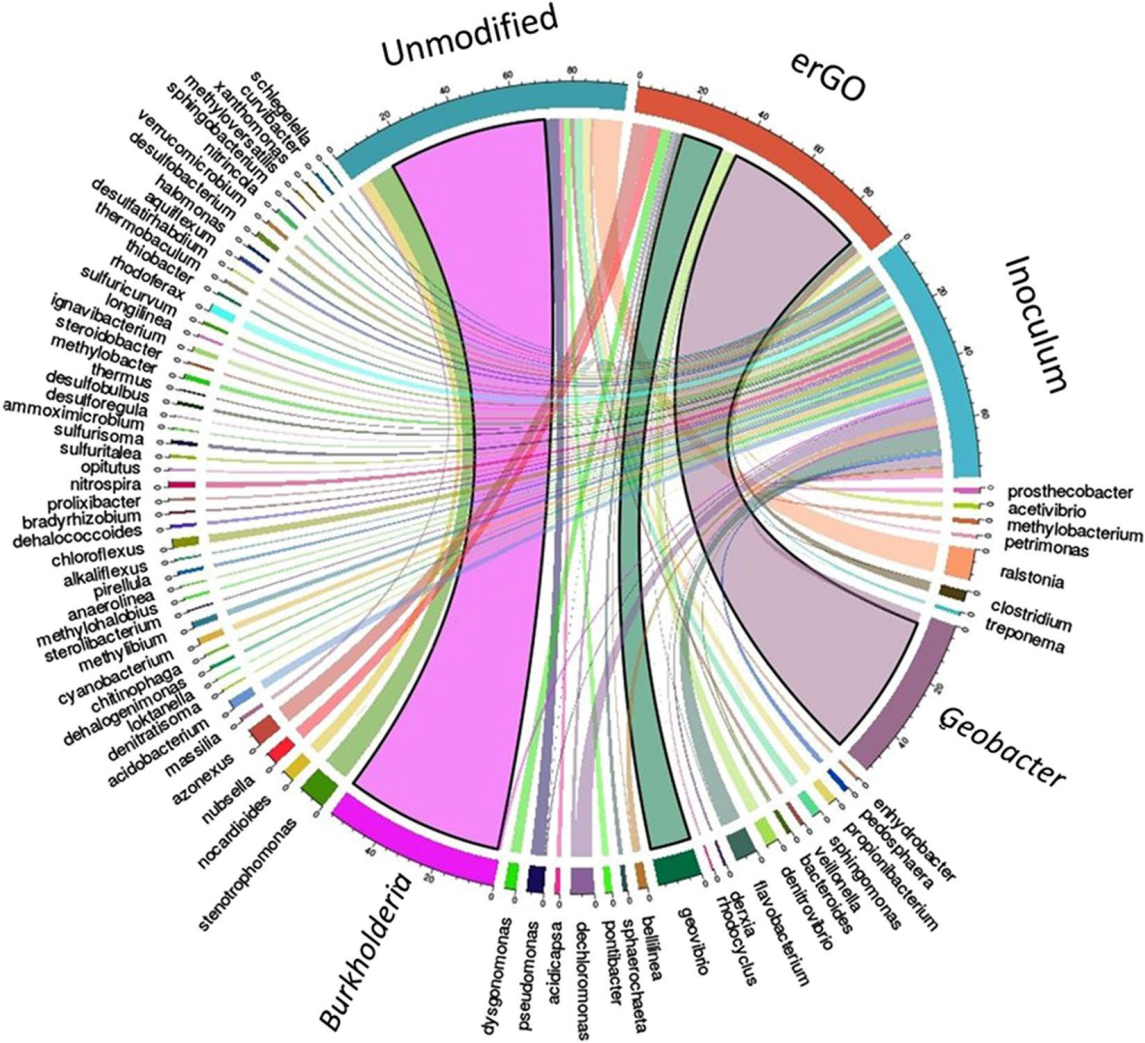
Chord diagram showing the genus that present a relative abundance more than 0.5% in the three samples analysed.

In order to gain insight into how the selective enrichment of electrochemically active bacteria in the anode is influenced by the modification process (GO electrodeposition), all genera above 0.5% are represented in a chord diagram (Fig. 8). Although the river mud used as inoculum is composed of many different genera in a very low relative abundance, a sharp specialisation is observed in both electrodes. Indeed, while in the unmodified electrode, *Burkholderiaceae* family was found at the highest frequencies (56% *Burkholderia* and 10% *Ralstonia*), the erGo electrode is dominated by the well-known exoelectrogenic *Geobacter* (50%), followed by other electrochemically active genera of interest in bioelectrochemical systems such as *Geovibrio* (14%), *Acetivibrio* (2%), *Denitrovibrio* (4%)^33^ and *Dysgonomonas* (4%).

Finally, we would like to point out that the observed increment of biomass formation in the reduced GO electrodes (see Table 1) is consistent with a recent work published by Virdis and Dennis^34^. However, while Virdis and Dennis^34^ did not find any enriched microbial taxa known to perform extracellular electron transfer (EET), our results demonstrate a clear enrichment in *Geobacter*, which seems to indicate that for certain exoelectrogens such as *Geobacter sp*, erGO acts as a selective agent over the environmental inoculum. Thus, the enhanced start-up of the GO reactors could be attributable not only to the improved electrochemical properties of the GO electrode (see section *Electrochemical behaviour of the erGO electrode*), but also to partially reduced functionalities and other network imperfections, that are characteristic of the graphene obtained via GO reduction and might explain the observed affinity for *Geobacter sp*. A related behaviour has been reported elsewhere^29^, where an enrichment in *Desulfomonas*, a marine exoelectrogen genus, was reached with a simultaneous GO bio-reduction. Therefore, our study is in agreement with others studies^29,35^, where the GO shows certain selectivity for some exoelectrogenic species.

### Outlook and future perspectives

Although the effect of the graphene oxide on the composition and development of bacterial communities in the anode of BES is still unclear, these results seem to confirm its beneficial impact on the enrichment and formation of a specific electroactive biofilm that enhances MEC performance by accelerating the start-up times.

The electrochemical deposition of graphene oxide, aside from improving the bio-electrochemical behaviour of the electrodes, offers some additional advantages, as it is a green, environmentally friendly, scalable and cost effective method. To this, the versatility in the handling of a liquid suspension must be added^6^. Still, the method presented here offers room for improvement. For instance, the selective enrichment effect of the GO observed in this study invites to tune up the C/O ratio during the reduction process^13^. This would allow researchers to influence the electrogenic biofilm population, which is an interesting possibility for biosensor development. Another research opportunity arises from the possibility of directly using electroactive microorganisms as bio-reduction agents in the electrode modification procedure, which is an idea already suggested in a recent publication^36^ and has the possibility to be integrated with the method proposed in this study.

## Conclusions

This article presents for the first time a single-step GO electroreduction procedure for the modification of carbon electrodes to be used in a in MEC. CVs performed in abiotic conditions revealed that the deposited graphene enhanced the heterogeneous electron transfer between the solution and the solid electrode. When operated as the anode of an MEC, the GO modified electrode accelerated the start-up and current stabilisation periods, probably as a result of its increased hydrophilicity. After 3 months of operation, both cells displayed a nearly similar behaviour in terms of current production, revealing that the performance-enhancing role of graphene can be circumscribed to the start-up process. Overall, the electrode modification seems to facilitate the formation of a robust biofilm, a selective enrichment and shows an affinity of the electrochemically active bacteria *Geobacter* for the erGO electrode, discarding any antibacterial effect, at least for this genus.
